# The Book World of Medicine and Science

**Published:** 1901-10-05

**Authors:** 


### The Book World of Medicine and Science.

Diseases of the Nervous System: A Text-book for Students and Practitioners of Medicine. By H. Oppenheim, M.D. Authorised translation by Edward E. Mayer, A.M., M.D. (London: J. B. Lippincott and Co., 1901. Price 21s. net.)

We have read this book with much interest. As a student's book it is somewhat large, but as a book for the practitioner who wishes to refer to it for the elucidation of individual cases, its pages will be found none too numerous, nor will its many illustrations turn out to be more than are required for the purpose intended. The first 90 pages of the book are devoted to the methods of examination which are employed in endeavouring to arrive at a true knowledge of the condition of the nervous system of a patient. This part is very freely illustrated, and the reader obtains a good idea of the extent to which an investigation of the nervous system may be carried by taking careful note of the condition and the functional activity of the various organs—motor and sensory areas—over which its periphery is distributed. Among other points we note that the author insists that "he who does not understand the use of an ophthalmoscope is no neurologist," in which he is no doubt right, for the retina is the one little bit of the nervous system that we can actually see. In the section dealing with the laryngeal muscles we fail to understand why the author has turned all his illustrations of the laryngeal opening upside down. Of course, if they are taken as representing what we should see if the man's head were cut off, and we looked directly down into his larynx, they are all right, but so long as the mirror is included in the picture, the view should be given as it is really seen, and as it is given generally by writers on laryngology. In classifying diseases of the spinal cord the author divides them into systemic diseases and diffuse disease, the latter including those which are due to diseases of the vertebra and the meninges, so that they are a rather mixed multitude. There is a good deal that is still doubtful in regard to all these maladies, but the story of the various conditions is well told. Perhaps the attempt to isolate and differentiate conditions which in fact tend much to run into one another is here and there a little strained, but that can hardly be helped. Until one recognises types one can hardly appreciate variations from them, and it really is by variations from type that the clues to causes and our most useful hints in regard to therapeutics are in some cases obtained. Diseases of the brain are well described, and most of our present knowledge regarding localising symptoms are given with considerable exactitude. As for the poor cerebellum it is dismissed in about four pages which, after the two hundred and seventy devoted to the brain itself, seems rather a small allowance. Perhaps the best bit of the book is the part in which the general symtomolcgy of cerebral diseases is described. When we come to the individual diseases the clinical pictures, although more detailed, are less convincing. Under the neuroses we find a rather wide group of diseases including hysteria and epilepsy, neurasthenia, and various forms of spasm, convulsive tic, &c., but not neuralgia, which comes under diseases of the peripheral nerves. Then we have diseases of the sympathetic system, angioneuroses, and tropho-neuroses, and lastly, toxic conditions with predominating nervous symptoms. Take the book altogether, it may be accepted as a full and complete account of diseases of the nervous system. It is not well divided into chapters, and to any reader who wishes to refer backwards and forwards it is a great aggravation to find hundreds of pages consecutively headed "diseases of the brain" or "diseases of the spinal cord," instead of the headlines being made to afford some notion of the contents of the page. The book, however, is very well printed and got up.

The Penalty of Death: or the Problem of Capital Punishment. By Josiah Oldfield. (London: George Bell and Sons. 1901. Price 3s. 6d. net.)

Dr. Oldfield has the knack of writing; and certainly this book is well written. Moreover, there is much in it that will excite the sympathy of many, especially of those who have faith in man and believe that his crimes are but the almost natural results of his surroundings. The whole drift of the book, however, which, of course, is a protest against the death penalty, seems to us to be hardly in touch with the actualities of life, and more calculated to impress one with the wrongness of things in general than to suggest any practicable means of putting them to rights. When the author proposes "hospitals for criminals," places in which these interesting people are to be "loved" back to spiritual health by "spiritual physicians," or in plain English by chaplains of a superior type, he seems to be the victim of some—shall we say, misconception? as to the nature of crime and the tendencies of criminals. But, in truth, the author is a man of curiously wide sympathies, in illustration of which we may quote what he says about anarchists. Referring to certain opinions as to the proper way of dealing with Anarchism he says: "For my own part, I have too much respect and reverence for a creed begotten of the evils of a repressive autocracy to think that this opinion correctly gauges the earnestness, the sincerity, the unselfishness of the leaders of anarchy." Such a writer is hardly the man to whom one would look for guidance in regard to the proper punishment of crime.

Surgical and Applied Anatomy. By Sir Frederick Treves, K.C.V.O., C.B., F.R.C.S. New Edition, revised by the Author, with the assistance of Arthur Keith, M.D., F.R.C.S. (London: Cassell and Co. 1901. Price 9s.)

It may perhaps be regarded as a work of supererogation to say much in regard to such a book as this, which has gone through several editions and has arrived at its twenty-fourth thousand. But although we have little to criticise we must not pass over in silence the issue of this new edition of so interesting a work, nor must we omit to recommend every student of anatomy to refer to its pages whenever he becomes tired of the work of the dissecting-rooms, for what this book does more successfully than most others which touch upon anatomical subjects is to give life and interest to the dead bones and flaccid muscles, and to show the importance and the practical applicability of the anatomical knowledge the acquisition of which sometimes becomes so wearisome. As a work of reference the book is useful and instructive; but it is as a means of showing to the student the true meaning of the dull facts of anatomy that it will be most profitably employed, and for this reason each section should be read by the student as soon as the "part" has been dissected. The occasional reading of a chapter of "Applied Anatomy" may be strongly recommended to the practitioner who fears that he may be getting rusty in his elementary subjects.

Golden Rules of Hygiene. By F. J. Waldo, M.A., M.D.(Cantab), D.P.H., Barrister-at-Law. (Golden Rules Series No. X. Bristol: John Wright and Co. Price 1s.)

Here we have another little book of "golden rules," this time on hygiene. So far as they go the rules given are good, but naturally they do not go very far, and criticism is disarmed by the fact that it is by no means clear whether they are intended for the instruction of students, practitioners, inspectors of nuisances, or the general public. Sometimes the endeavour to be short leads to curious results, as for example in the following aphorism, "Observe that the aim of modern sewers is to be water-tight."

